# Structure of the Human Discs Large 1 PDZ2– Adenomatous Polyposis Coli Cytoskeletal Polarity Complex: Insight into Peptide Engagement and PDZ Clustering

**DOI:** 10.1371/journal.pone.0050097

**Published:** 2012-11-19

**Authors:** Kevin C. Slep

**Affiliations:** Department of Biology, University of North Carolina at Chapel Hill, Chapel Hill, North Carolina, United States of America; University of Oulu, Finland

## Abstract

The membrane associated guanylate kinase (MAGUK) family member, human Discs Large 1 (hDlg1) uses a PDZ domain array to interact with the polarity determinant, the Adenomatous Polyposis Coli (APC) microtubule plus end binding protein. The hDLG1-APC complex mediates a dynamic attachment between microtubule plus ends and polarized cortical determinants in epithelial cells, stem cells, and neuronal synapses. Using its multi-domain architecture, hDlg1 both scaffolds and regulates the polarity factors it engages. Molecular details underlying the hDlg1-APC interaction and insight into how the hDlg1 PDZ array may cluster and regulate its binding factors remain to be determined. Here, I present the crystal structure of the hDlg1 PDZ2-APC complex and the molecular determinants that mediate APC binding. The hDlg1 PDZ2-APC complex also provides insight into potential modes of ligand-dependent PDZ domain clustering that may parallel Dlg scaffold regulatory mechanisms. The hDlg1 PDZ2-APC complex presented here represents a core biological complex that bridges polarized cortical determinants with the dynamic microtubule cytoskeleton.

## Introduction

Cell polarity is a core, biological process requisite for cell division, chemotaxis and multi-cellular development. Polarity factors integrate cellular cues and translate these signals into directed cytoskeletal change. This requires an interplay between polarity determinants and the cytoskeleton, an association that is inherently complicated by membrane and cytoskeletal dynamics. To stably link cortical determinants with the dynamic cytoskeleton, a bridge must be established that retains membrane association while sustaining a static or processive cytoskeletal attachment. One example of a molecular bridge between the membrane and the microtubule cytoskeleton is the complex formed between Dlg1 and APC, a protein that binds polymerizing microtubule plus ends in an EB1-dependent manner [1], [2], [3], [4], [5], [6].

**Figure 1:**
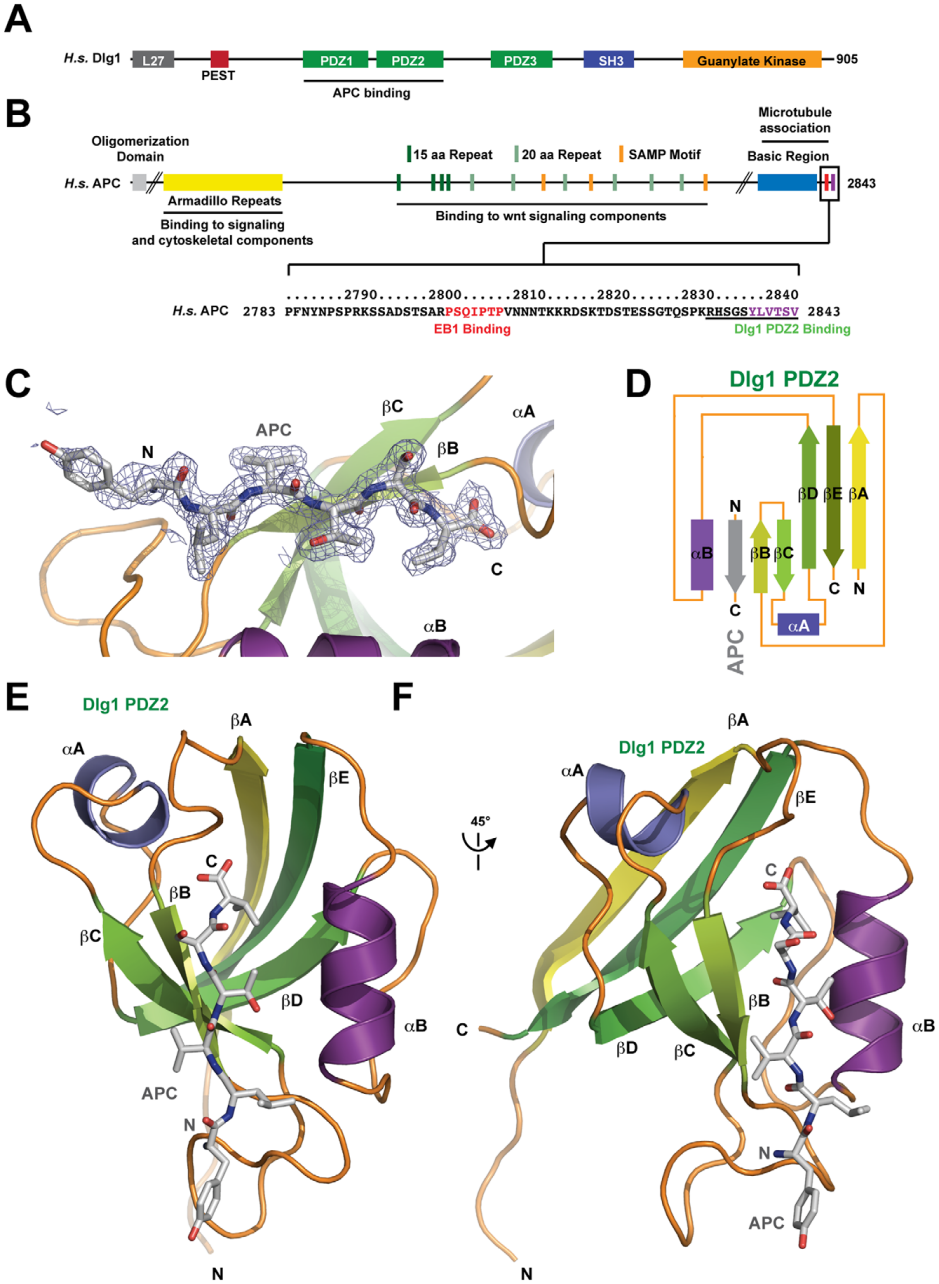
Overall structure of the Dlg1 PDZ2-APC complex. A) Domain arrangement of H.s. Dlg1. Shown is the L27 domain (grey), PEST motif (red), PDZ domains 1–3 (green), SH3 (dark blue), and guanylate kinase domain (orange). B) Domain arrangement of H.s. APC. Shown is the oligomerization domain, the armadillo repeat domain, 15 and 20 aa repeat sequences used in β-catenin binding, SAMP motifs used for axin binding, a basic region with microtubule binding activity, and C-terminal EB1 and Dlg1 binding sites. The C-terminal region is boxed and corresponding sequence shown below. Highlighted in red is the SxIP motif and flanking residues that bind the microtubule plus end tracking protein EB1. The C-terminal six residues that bind Dlg1 PDZ2 are highlighted in purple. The APC peptide synthesized in this study is indicated by an underline. C) Zoom view of the APC peptide bound to hDlg PDZ2. Final, refined 2Fo-Fc electron density around the APC peptide is displayed and contoured at 1.0 σ. D) 2° structure topology of Dlg1 PDZ2 complexed with the C-terminal APC β-strand (grey), which runs anti-parallel to Dlg1 PDZ2 βB and parallel to αB. E) Ribbon diagram of Dlg1 PDZ2 complexed with the APC C-terminal six residues shown in stick format. F) Structure shown in E, rotated 45° about the y-axis. The Dlg β-strands are colored in a yellow to dark green continuum, loop regions are colored orange, helices αA and αB are colored slate and purple respectively, and the APC peptide is colored grey (C–F).

Dlg1 is a MAGUK protein family member localized to polarized structures including epithelial junctions, stem cell cortical regions, and neuronal synapses [7], [8], [9], [10]. MAGUK proteins adhere to a common domain architecture comprising one to three PDZ domains that bind target proteins, an SH3 domain, a variable HOOK sequence, and an enzymatically inactive guanylate kinase domain (Figure 1A) [11], [12], [13]. Collectively, MAGUK protein domains operate as scaffolding that actively cluster and regulate the components they bind. Canonical PDZ-protein interactions involve PDZ domain binding to a target protein’s C-terminal region [14]. PDZ domains are classified based on the target sequences they bind. Common PDZ targets are classified as type I: -S/T-X-φ, type II: -φ-X-φ, or type III: -D/E-X-φ motifs where φ is a valine or leucine. Dlg1 contains three PDZ domains, classified as G-H PDZ domains with specificity for type I PDZ-binding motifs [15]. PDZ1 and PDZ2 directly abut each other while a 49 amino acid linker bridges PDZ2 and PDZ3. Intra-molecular PDZ domain interactions have been observed in ligand-free PDZ domain-containing proteins including PSD-95, GRIP, and syntenin [16], [17], [18], [19], [20], [21]. Additional studies have demonstrated ligand-dependent changes in intra-molecular PDZ domain interactions, specifically, the release of PDZ interactions upon ligand binding [22]. But how PDZ domain arrays promote target clustering remains an outstanding question. PDZ-mediated clustering enables the MAGUK proteins to coordinate and modulate signaling and cytoskeletal components in space and time. Mapping the diverse modes PDZ domains use to bind target proteins and interact with neighboring PDZ domains is fundamental to understanding how MAGUK proteins operate. Factors found to bind the Dlg1 PDZ domains include the Ca^2+^pump 4b, p56lck tyrosine kinase, human papillomavirus E6 protein, and the polarity determinant APC [1], [23], [24], [25]. Dlg1 is competent to bind the APC C-terminus using either PDZ1 or PDZ2. The APC C-terminus is a type I PDZ binding motif that binds Dlg1 PDZ1 and PDZ2 with 18 µM and 1 µM affinity, respectively [26].

APC is a conserved, multi-domain, multi-motif scaffolding protein involved in β-catenin destruction, cell adhesion, microtubule dynamics, and cytoskeletal signaling [27], [28], [29], [30], [31], [32], [33]. Mutations in APC have pleotropic effects including aberrant wnt signaling, defects in cell adhesion and adherens junction structure, developmental defects, and colorectal cancer [34], [35]. Underlying its multifunctional cellular role is its diverse domain architecture that spans 2843 amino acids. Central motifs, designated 15 and 20 amino acid repeats and SAMP motifs are determinants that bind wnt signaling components and orchestrate β-catenin destruction [36], [37]. Delineated cytoskeletal binding determinants flank the central motifs involved in wnt-signaling. An N-terminal armadillo repeat domain binds to cytoskeletal and signaling components including the kinesin KAP3, the guanine nucleotide exchange factor ASEF, and the GTPase-activating protein IQGAP [33], [38], [39]. The APC C-terminus contains a basic region that binds microtubules as well as a SxIP-motif that binds the microtubule plus end tracking protein EB1, localizing APC to the polymerizing microtubule plus end [40], [41], [42]. The APC SxIP motif is 30 amino acids N-terminal to the Dlg1 PDZ-binding motif (Figure 1B).

The microtubule cytoskeletal array is polarized, with minus ends predominantly focused at a microtubule organizing center. Microtubule plus ends fan out towards the cell periphery and are the focal points of microtubule dynamic instability [43]. Accordingly, a host of factors associate with the microtubule plus end to regulate its dynamics. A prime factor associated with the polymerizing microtubule plus end is EB1 [4]. The EB1 N-terminal calponin homology domain recognizes the GTP state of the polymerizing microtubule, thereby binding microtubule lattice determinants specific to the polymerizing tip [44], [45]. The conserved EB1 C-terminal dimerization domain recruits numerous proteins to the microtubule lattice by binding a short amino acid motif termed a SxIP motif [42]. SxIP motif-containing proteins are functionally diverse, and include polarity determinants, cytoskeletal crosslinkers, signaling molecules and regulators of microtubule dynamics. The adenomatous polyposis coli (APC) tumor suppressor has a C-terminal SxIP motif that affords EB1-dependent APC microtubule plus end tracking activity. Serendipitously, EB1 was first identified in a yeast two-hybrid screen for APC binding proteins, and was named EB1 to reflect its ability to bind the end of APC; subsequently it was identified as a microtubule plus end binding protein [3], [4].

Collectively, the Dlg1-APC-EB1 complex mediates a dynamic link between polarized membrane regions and the microtubule cytoskeleton [2], [6]. APC can use its basic region to bind microtubules directly, or its SxIP motif to bind EB1, which localizes it to the polymerizing microtubule plus end. The high-affinity Dlg1-APC link is mediated by PDZ2. While high-resolution structural information is available for the Dlg1 PDZ1-APC interaction, only a partially occupied Dlg1 PDZ2-APC structure has been determined that showed a splayed APC peptide, engaging PDZ2 using only its ultimate four residues [26]. When compared to the Dlg1 PDZ1-APC structure that binds the ultimate five APC residues, the PDZ2-APC structure raised questions as to why the PDZ2-APC interaction was an order of magnitude greater than the PDZ1-APC interaction. Here I report the crystal structure of the Dlg1 PDZ2-APC complex determined to a resolution of 2.0 Å. This structure shows an extensive interaction between PDZ2 and APC that spans the ultimate six APC residues and highlights additional binding determinants that likely promote the PDZ2-APC interaction. This structure provides molecular details on the Dlg1-APC polarity complex as well as determinants that may underlie MAGUK protein dynamic scaffolding and target clustering.

## Materials and Methods

### Cloning and Purification

DNA encoding human Dlg1 PDZ2, residues 310–407, was generated by PCR sewing using oligonucleotide templates with optimal *E. coli* codon usage. The Dlg1 PDZ2 fragment was inserted into pGEX-2T (GE Healthcare) forming a thrombin-cleavable, N-terminal GST fusion construct. BL21 DE3 pLysS *E. coli* were transformed with pGEX-2T-Dlg1 PDZ2, grown in LB media under 50 µg/L ampicillin selection at 37°C to an optical density at 600 nm of 1.0 at which point protein expression was induced by the addition of 0.1 mM isopropyl-1-thio-β-D-galactopyranoside (final concentration), the temperature was lowered to 20°C, and induction proceeded for 16 hours. Cells were harvested by centrifugation at 2100×g for 10 minutes and resuspended in 200 mL buffer A: 25 mM Tris pH 8.0, 300 mM NaCl, 0.1% β-mercaptoethanol, and lysed by iterative rounds of sonication at 4°C followed by sample cooling. 0.2 mM phenylmethylsulfonyl fluoride (final concentration) was added during sonication and the lysate clarified by centrifugation at 23,000×g for 45 min. The supernatant was loaded onto a 10 ml Glutathione Sepharose Fast Flow affinity column (GE Healthcare). GST-Dlg1 PDZ2 was eluted from the glutathione column with 50 ml of 25 mM glutathione pH 8.0 in buffer A. 1 mM CaCl_2_ was added to the eluate, as was 25 µL of a 1 mg/mL thrombin stock (Heamatologic Technologies) to cleave GST from Dlg1 PDZ2, leaving an N-terminal gly-ser cloning artifact on the PDZ2 domain. Proteolysis proceeded for 12 hours at 4°C. Thrombin was removed from the sample by filtering the sample over 0.5 mL Benzamidine Sepharose (GE Healthcare). The sample was exchanged into 100 mL buffer B (25 mM HEPES, pH 6.8 and 0.1% β-mercaptoethanol) using an Amicon Ultra 3 kDa spin concentrator (Millipore) and loaded onto a 10 mL SP Sepharose Fast Flow column (GE Healthcare) and eluted using a linear 0–1 M NaCl gradient in buffer B. Dlg1 PDZ2 peak fractions were pooled and exchanged into 100 mM NaCl, 25 mM Tris pH 7.0, and 0.1% β-mercaptoethanol using an Amicon Ultra 3 kDa spin concentrator (Millipore), concentrated to 25 mg/mL, snap frozen in liquid nitrogen and stored at −80°C. All purification procedures were executed at 4°C.

### Synthesis of the APC C-terminal Peptide

An APC C-terminal peptide encompassing residues RHSGSYLVTSV (2833–2843) was synthesized with an N-terminal tetramethyl rhodamine (TMR) tag by the Yale Keck Biotechnology Resource Laboratory, purified by HPLC and verified by mass spectrometry.

### Crystallization

1.4 mM Dlg1 PDZ2 was incubated with 1.7 mM APC C-terminal peptide in 100 mM NaCl, 25 mM Tris, pH 7.0, and 0.1% β-mercaptoethanol for one hour on ice. Crystallization followed the hanging drop protocol using 2 µL of the Dlg1 PDZ2-APC mixture and 2 µL of a 1 mL well solution containing 200 mM sodium acetate, 100 mM sodium cacodylate pH 6.25, 28% (w/v) polyethylene glycol 8000. Thin crystal sheets grew at 20°C to dimensions of 20×100×600 µm over the course of a week. Crystals displayed an enhanced red color relative to the surrounding solution, indicative of TMR-APC peptide incorporation into the crystals. Crystals were transferred to cryoprotectant containing well solution supplemented with 30% glycerol and flash frozen in liquid nitrogen.

### Data Collection, Structure Determination, and Refinement

Dlg1 PDZ2-APC crystals were maintained at 100 K under a cryo-cooled nitrogen stream and diffraction data collected at the Advanced Light Source synchrotron beamline 8.3.1. Data were indexed, integrated and scaled using HKL2000 [46] (Table 1). The structure was determined using the AutoMR molecular replacement program (PHENIX crystallographic suite) and a model of the human apo Dlg3 PDZ2 domain (pdb accession code 2FE5). One solution was found in the asymmetric unit. The molecular replacement solution showed clear electron density for the APC peptide. The model was built using AutoBuild (PHENIX) [47] and refined iteratively through manual builds in Coot [48] followed by refinement runs using phenix.refine (PHENIX) against a maximum likelihood target function. Refinement statistics were monitored using a Free R, calculated using 10% of the data, randomly excluded from refinement. The final model includes one Dlg1 PDZ2 molecule (chain A: residues 310–406, residue R310 modelled as alanine), one APC molecule (chain B: residues 2838–2843), and 106 water molecules. Structure figures were produced using PyMOL (http://pymol.sourceforge.net).

**Table 1:** Data Collection and Refinement Statistics.

Data Collection	
Wavelength (Å)	1.127
Space group	P2_1_2_1_2_1_
Cell dimensions: a,b,c (Å)	27.1, 51.2, 71.8
Resolution (Å)	70–2.00 (2.07–2.00)
Reflections: Measured/Unique	32635/6846
Completeness (%)	97.8 (82.9)
Mean redundancy	4 (3)
<I/σI>	20.7 (4.8)
R_sym_ a	0.07 (0.22)
**Refinement**	
Resolution (Å)	29.4–2.00 (2.15–2.00)
R^b^/R_free_ (%)^c^	17.1 (16.4)/18.3 (18.7)
Reflections: R/R_free_	6158/688
Total atoms: Protein/Water	769/106
Stereochemical ideality (rmsd): Bonds/Angles (Å/°)	0.007/0.980
Mean B-factors (Å^2^): Protein/Water	15.9/25.3
Ramachandran analysis: Favored/Allowed (%)	97.0/3.0
Parentheses list statistics for the high resolution shell	

a R_sym_ = ∑|I_i_−<l>|/∑I where *I_i_* is the integrated intensity of the i-th observation and<I>is the mean intensity of the reflections over Friedel and symmetry equivalents.

b R value = ∑(|F_obs_|−k|F_calc_|)/∑|F_obs_|.

c R_free_ is calculated using a 10% subset of the data that are removed randomly from the original data and excluded from refinement.

### Data Deposition

Coordinates for the human Dlg1 PDZ2-APC complex have been deposited in the Protein Data Bank under accession code: 4G69.

## Results

### Structure Determination of the Human Dlg1 PDZ2-APC Complex

To examine the molecular basis for the Human Dlg1 PDZ2– APC interaction, the Dlg1 PDZ2 domain (residues 310–407) was purified and complexed with a peptide corresponding to the C-terminal 11 residues of APC with an N-terminal TMR fluorophore covalently bound. A 1∶1.2 molar ratio of the PDZ domain:APC peptide was used. Thin crystal plates grew over the course of a week from a polyethylene glycol condition at pH 6.25. Crystals were bright red compared to the surrounding solution, indicative of peptide incorporation into the crystal. Diffraction data was collected to a resolution of 2.0 Å and processed in the space group P2_1_2_1_2_1_. A peptide-free human Dlg3 PDZ2 model (SAP97, PDB code 2FE5) was used to find one molecule in the asymmetric unit. Strong electron density was evident in the PDZ peptide-binding site and the final six C-terminal residues of the APC peptide could be assigned and built into the electron density. The structure was built and refined to final R and R_free_ factors of 17.1% and 18.3%, respectively. No electron density was evident for the TMR fluorophore or the N-terminal five amino acids of the APC peptide (Figure 1C). The full PDZ domain was modelled, including residues 310–406.

### Overall Architecture of the Dlg1 PDZ2-APC Complex

Dlg1 PDZ2 is a canonical PDZ domain fold (Figure 1C–F) [49]. A twisted, anti-parallel β-sheet establishes the core of the domain and progresses through five β-strands: βA-βE-βD-βC-βB. The βB-βC segment is connected to βD by a short, single turn helix, αA that packs against βA, covering the hydrophobic core. A second helix, αB, with its flanking loop regions, connects βD and βE. The loop bridging βD and αB is packed between these structural elements and runs anti-parallel to them. Collectively, the domain forms a compact structure with an exposed, hydrophobic groove framed by βB and αB as well as the extended βA-βB and βB-βC loops. Nestled in the hydrophobic groove is the APC peptide in a β-strand conformation, running anti-parallel to the adjacent βB strand and parallel to the αB helix. The APC C-terminal carboxylate group is positioned proximal to the βA-βB loops. Distal to this peptide-binding groove, the PDZ domain’s N- and C-termini are positioned next to one another.

### Species- and Dlg Isoform-specific Conserved Rims Frame the APC Binding Site

**Figure 2:**
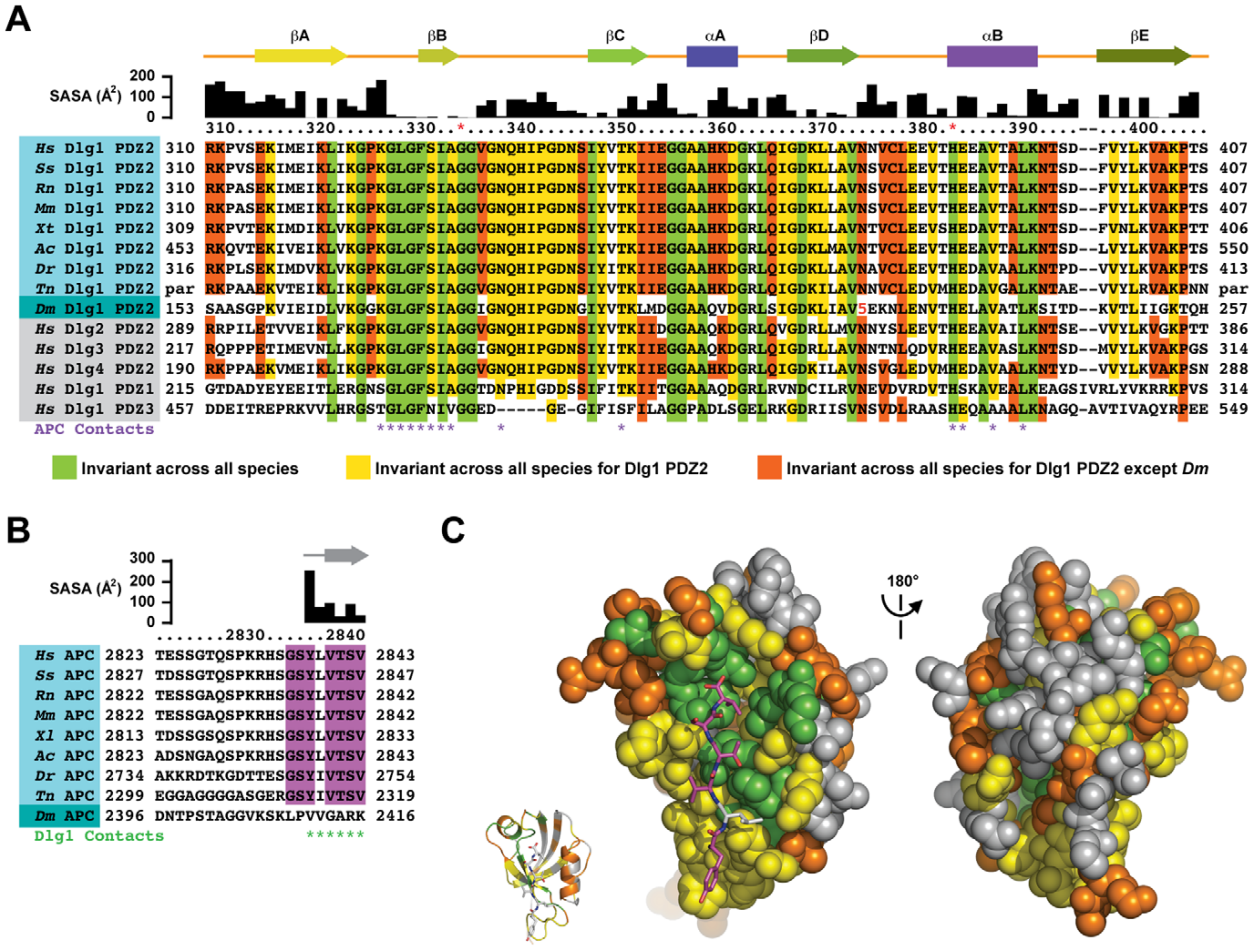
The Dlg1 PDZ2 peptide-binding cleft is highly conserved. A) Alignment of PDZ domains from human Dlg1 as well as PDZ2 from human Dlg2-4 and Dlg1 from Sus scrofa (Ss), Rattus norvegicus (Rn), Mus musculus (Mm), Xenopus tropicalis (Xt), Anolis carolinensis (Ac), Danio rerio (Dr), Tetraodon nigroviridis (Tn), and Drosophila melanogaster (Dm). Only a partial sequence was available for Tn, thus residue numbers are not listed. The alignment was generated manually through analysis of conserved residues. Solvent accessible surface area for each residue in the Dlg1 PDZ2-APC complex is indicated above the alignment as is secondary structure. Residues numbers above the alignment are for human Dlg1 PDZ2. Red asterisks indicate the two sites commonly used to classify PDZ domains. Dlg1 PDZ2 residues that contact APC are indicated. Residues invariant across all PDZ domains are colored green. Residues invariant across all Dlg1 PDZ domains are colored yellow. Residues invariant across all Dlg1 PDZ2 domains except Drosophila are colored orange. B) Alignment of APC C-terminal 21 amino acids for species aligned in A. The alignment was generated manually through analysis of conserved residues. Residues invariant across all species except Drosophila are colored purple. Solvent accessible surface area, secondary structure, and residues that contact Dlg1 PDZ2 are indicated. C) Structure of the Dlg1 PDZ2-APC complex with Dlg1 PDZ2 shown in spherical format and the APC peptide shown in stick format. Invariance is colored according to the alignments in A and B. The ribbon model shown in inset is in the same orientation as the adjacent spherical model. The two spherical models are related by a 180° rotation about the y-axis.

To investigate specificity in the Dlg1 PDZ2-APC interaction, I aligned Dlg1 PDZ2 domains from diverse species with human Dlg1 PDZ1-3 and Dlg2-4 PDZ2. Invariance was mapped with three criteria: those residues invariant across all PDZ domains aligned, those residues invariant across Dlg1 PDZ2 domains aligned, and those residues invariant across Dlg1 PDZ2 domains aligned but different in *Drosophila* Dlg1 PDZ2 (Figure 2A). *Drosophila* was set as a point of divergence given that *Drosophila* APC does not contain a C-terminal PDZ-binding motif. An alignment of APC C-terminal sequences from the same set of species used in the Dlg alignment is presented in Figure 2B. In contrast to *Drosophila* APC, all other species aligned ranging from human to pufferfish (*Tetraodon nigroviridis*) are invariant across seven of the ultimate eight APC residues, conforming to a type I PDZ binding motif (Figure 2B). When the three contours of Dlg PDZ invariance are mapped on the Dlg1 PDZ2-APC complex, a core set of residues, invariant across all PDZ domains aligned, is positioned in the peptide binding groove to mediate canonical PDZ-type I peptide interactions (Figure 2C, green) [49]. A signature, invariant PDZ motif: GLGF, underlies this site and forms contacts with the ultimate three APC residues, including the carboxylate group. Surrounding the groove are residues positioned at the peptide’s N- and C-termini that are invariant across all Dlg1 PDZ2 species aligned, including *Drosophila* (Figure 2C, yellow). However, flanking either side of the APC β-strand are residues invariant across the Dlg1 PDZ2 members aligned, except *Drosophila* (Figure 2C, orange). A number of these residues are also present in other human Dlg PDZ domains, including the Dlg1 PDZ1 domain that also exhibits APC C-terminus binding activity *in vitro*. Many of these invariant residues reside on the αB helix and are APC peptide binding determinants while others flank the peptide binding site, but are not directly involved in peptide binding. Overall, a collective, invariant site is defined by Dlg PDZ domains from species that contain the C-terminal APC sequence GSYφVTSV, where φ is a hydrophobic residue. While some of these residues are APC-binding determinants, flanking peripheral residues, along with the bound APC molecule, may define a composite Dlg1 PDZ2-APC binding site for other factors involved in Dlg1 scaffolding.

### The Dlg1 PDZ2-APC Binding Determinants

**Figure 3:**
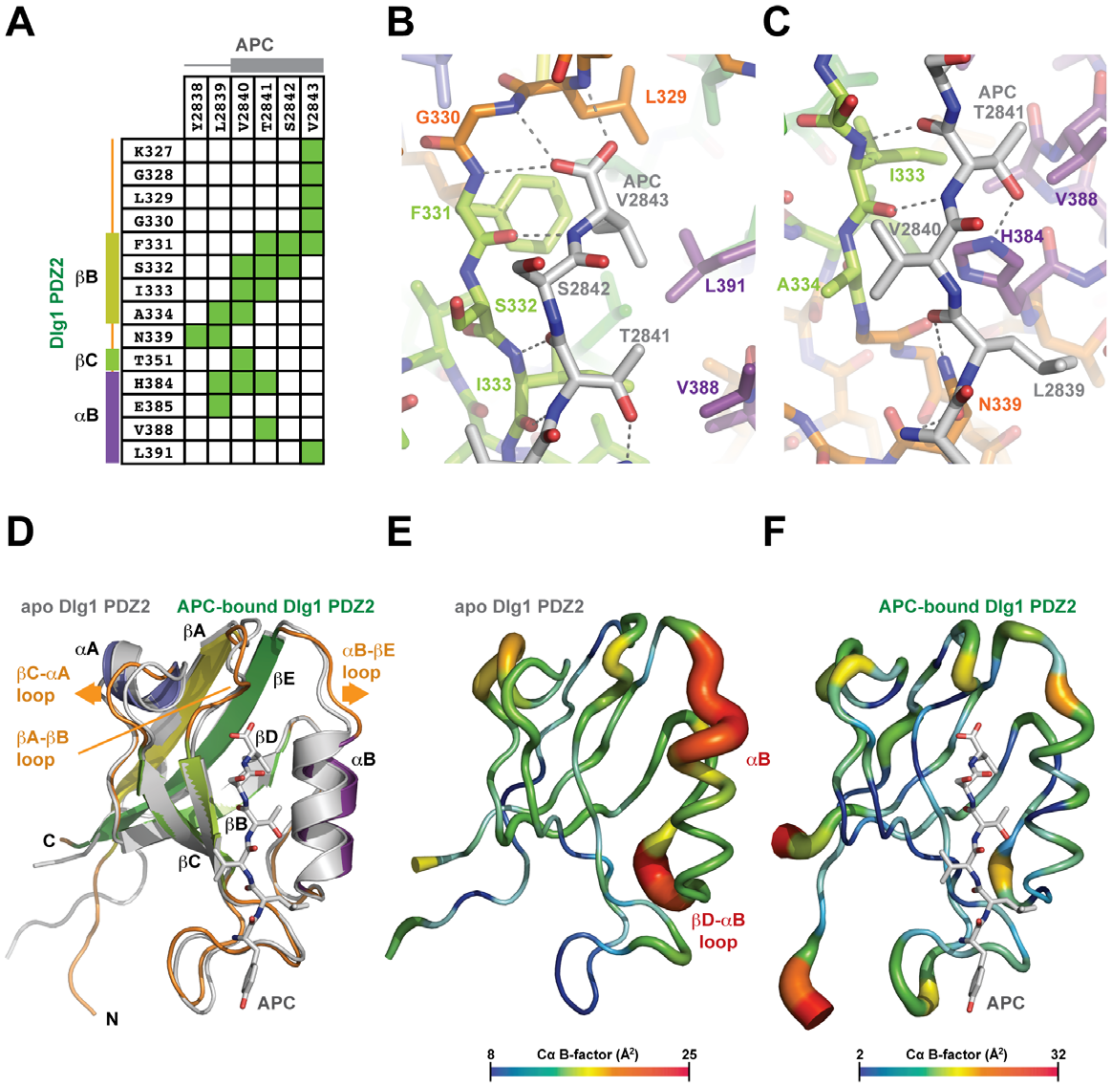
The Dlg1 PDZ2 domain binds core, conserved determinants in the APC C-terminus. A) Interaction matrix between Dlg1 PDZ2 (y-axis) and the APC C-terminal peptide (x-axis). Residue number and the corresponding secondary structure are indicated along the axes. Interactions (hydrogen bonds, salt bridges, and van der Waals) are contoured at a 4.5 Å cut-off. B, C) Stick model of the Dlg1 PDZ2-APC binding site. Dlg1 PDZ2 and APC are colored as in Fig. 1C. Hydrogen bonds involved in the Dlg1 PDZ2-APC interaction are indicated with dashed lines. D) Alignment of the Dlg1 PDZ2-APC structure (colored as in Fig. 1C) and the apo hDlg1 PDZ2 structure (grey, 98% sequence identity, includes I342W and C378A mutations outside the peptide binding region, engineered for measuring protein folding, PDB code 2X7Z [50]), highlighting outward movement in the αB helix. Orange arrows highlight structural differences. E, F) Comparative B-factor analysis of the apo Dlg1 PDZ2 structure and the Dlg1 PDZ2-APC structure aligned in D. Cα B-factor ranges are color-coded according to the key below each molecule.

The APC C-terminal peptide lies in the PDZ type I-specific furrow defined by βB and αB. The APC peptide forms a β-strand that makes a canonical hydrogen bond network with the anti-parallel βB strand (Figure 3A–C). The ultimate three APC residues: TSV, conform to the type I PDZ peptide motif: S/T-X-φ, and make standard contacts in the furrow. Specifically, the APC V2843 carboxylate group makes hydrogen bonds with the backbone amides of L329, G330, and F331 (Figure 3A,B). The APC V2843 side chain packs in the hydrophobic core of the furrow, making van der Waals contacts with L329, F331, and L391. The APC T2841 side chain hydroxyl makes a hydrogen bond to the H384 ring Nε while the T2841 Cβ and Cγ atoms make van der Waals contacts with I333 and V388 respectively (Figure 3C). While the ultimate APC residues conform to standard type-I PDZ interactions, the penultimate three APC residues: YLV, also engage the Dlg1 PDZ2 peptide binding furrow and make specific contacts with Dlg1. V2840 contributes to the anti-parallel β-strand interaction while the backbone spanning APC Y2838 and L2839 is stabilized by a hydrogen bonding network with the Dlg1 N339 carboxamide group (Figure 3C). APC L2839 and V2840 also make van der Waals contacts with Dlg1 residues H384 and A334 respectively. Collectively, Dlg1 PDZ2 and the APC peptide bury 744 Å^2^ of solvent accessible surface area.

### PDZ Domain Plasticity and Conformational Change

To investigate conformational change that occurs upon APC peptide binding, the Dlg1-PDZ2-APC structure was superimposed on the apo structure of human Dlg1 PDZ2 (Figure 3D) [50]. Distinct structural differences are noted in the peptide-binding cleft. In the apo structure, αB is closer to the βB strand than in the APC-bound structure, indicative that the peptide-binding cleft splays to allow APC access and binding. Structural changes in the APC-bound structure are also evident in the loop regions that flank the peptide binding site, these include the αB-βE loop, the βA-βB loop, as well as distal structural elements including the βC-αA loop which is shifted away from the peptide binding site, potentially through movement in the adjacent βA-βB loop. Comparative B-factor analysis for the apo and APC-bound PDZ2 structures show relatively high B-factors in regions that correlate with structural changes between the two states (Figure 3D–F). Normalizing the B-factor range for the two structures, the βB strand and the αB helix displayed very low B-factors in the Dlg1 PDZ2-APC structure in contrast to the Dlg1 PDZ2 apo structure where the highest B-factors mapped to the αB helix and the flanking loops. This indicates that the αB helix is moderately mobile and becomes stabilized once the APC peptide engages the furrow.

### High-occupancy Conformation of the APC C-terminal Region

**Figure 4:**
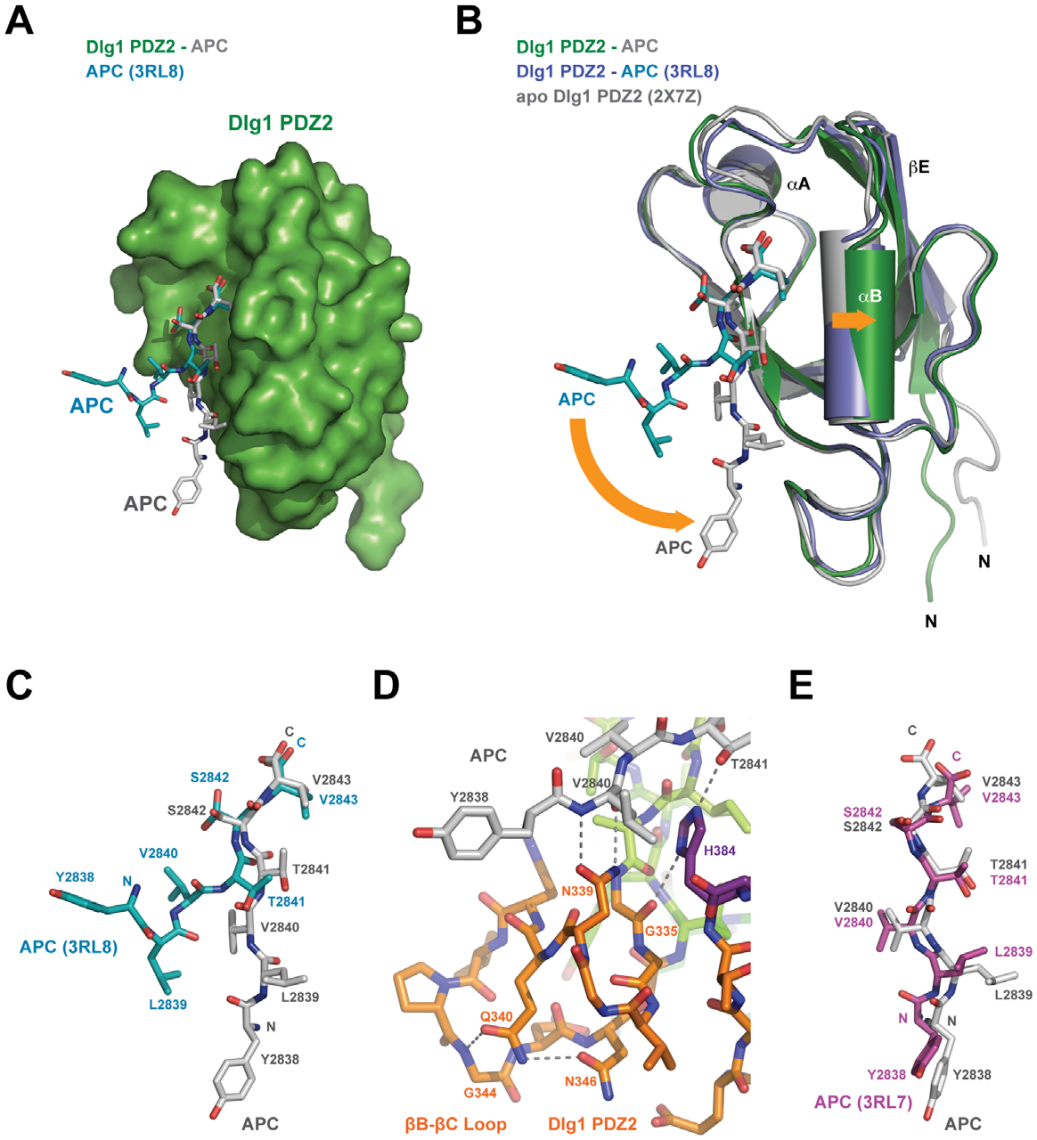
Differential APC peptide binding correlates with PDZ domain conformation change. A) The Dlg1 PDZ2-APC structure determined in this study with the PDZ2 domain shown in surface representation and colored green, APC peptide shown in stick format and colored grey. Superimposed is the structure of the APC peptide bound to Dlg1 PDZ2 as determined by Zhang et al. (PDB code 3RL8 [26]). The 3RL8 APC peptide is shown in stick format and colored cyan; the PDZ2 domain is not shown for simplicity. B) Ribbon diagram of the Dlg1 PDZ2-APC complex determined in this study (Dlg1 PDZ2 in green, APC in grey), with the structure of the Dlg1 PDZ2-APC complex determined by Zhang et al. (Dlg1 PDZ2 in slate, APC peptide in cyan, PDB code 3RL8) and the apo Dlg1 PDZ2 structure (grey, PDB code 2X7Z [50]) superimposed. The conformation of the Dlg1 PDZ2 domain bound to APC determined by Zhang et al. is more homologous to the Dlg1 PDZ2 apo structure than to the Dlg1 PDZ2-APC structure determined in this study, specifically in the positioning of αB and the βC-αA loop that move outward from the peptide binding cleft upon APC binding. Conformational differences between the PDZ domains and the APC peptides are highlighted by orange arrows. C) Comparison of the APC peptide bound to Dlg1 PDZ2 from Zhang et al. versus this study, oriented as shown in A. The structure of the APC peptide determined in this study use all residues shown to bind the Dlg1 PDZ2 structure. Significant repositioning occurs over residues L2839-T2841 as well as the side chain rotamer positioning of S2842. D) Zoom view of the APC peptide N-terminal region and its interaction with the βB-βC loop. APC and Dlg1 PDZ2 are shown in stick format, colored as in Figure 1C. The backbone region of APC V2840 is stabilized by hydrogen bonds with the N339 side chain. Specific hydrogen bonds are shown as dashed lines. E) Orientation of the APC peptide bound to Dlg1 PDZ2 determined in this study (shown in grey, stick format) versus the APC peptide structure bound to Dlg1 PDZ1, chain J, determined by Zhang et al. (PDB code 3RL7 [26]), colored purple and shown in stick format. APC peptides are shown alone, positioned after structurally aligning the respective PDZ domains they are bound to (not shown).

Recent work by Zhang et al. analyzing the structure and binding mode of Dlg1 with APC determined a significantly different structural engagement between Dlg1 PDZ2 and APC [26]. This crystal structure, determined in space group P2_1_, contained five Dlg1 PDZ2 molecules in the asymmetric unit, of which only one PDZ molecule showed electron density into which an APC peptide could be modelled. In contrast, the structure presented here, determined in space group P2_1_2_1_2_1_, contains a single Dlg1 PDZ2 molecule in the asymmetric unit, with a fully-bound, APC C-terminal peptide (Figure 4A). When the Dlg1 PDZ2-APC complex by Zhang et al. is compared to the structure presented here as well as the Dlg1 PDZ2 apo structure, the orientation of the APC peptide and the PDZ peptide binding furrow adopt highly different conformations (Figure 4B, orange arrows). First, the APC peptide in the current structure docks along the entire PDZ peptide-binding furrow, forming an extensive hydrogen bonding network with Dlg1 PDZ2, extending the PDZ β-sheet through anti-parallel strand extension and burying 372 Å^2^ of the peptide’s solvent accessible surface area. In contrast, the APC C-terminal peptide in the Zhang et al. structure only engages the PDZ furrow using its C-terminal four residues. Between the two structures, the ultimate C-terminal residue, V2843, is positioned in the furrow equivalently. However, every preceding APC residue in the Zhang et al. structure adopts a significantly different spatial conformation in regard to both the backbone position as well as side chain rotamer conformation. In the Zhang et al. structure, the APC peptide N-terminal region is displaced from the PDZ domain, splayed nearly 45° from the PDZ domain leaving only the ultimate four APC residues to bind Dlg1 PDZ2 (Figure 4A–C). This contrasts with the ultimate six APC residues that engage Dlg1 PDZ2 in the structure reported here. The two structures also differ with regard to the conformation of the PDZ domain’s peptide-binding furrow. In the Zhang et al. structure, the Dlg1 PDZ peptide-binding furrow is structurally more akin to the peptide-free Dlg1 PDZ2 apo structure, where αB is positioned closer to βB, clamping the peptide furrow (Figure 4B) [50]. In the Dlg1 PDZ2-APC structure presented here, αB is displaced, opening the PDZ peptide-binding furrow, enabling APC to bind the length of the furrow. Collectively, the differential binding states observed in the two Dlg1 PDZ2-APC structures likely reflect steps along a binding pathway: initial binding via canonical interactions between the ultimate C-terminal valine and the PDZ domain as observed in the Zhang et al. structure, followed by docking of the N-terminal residues into the furrow and displacement of the αB helix as observed in the structure reported here.

Zhang et al. predicted that APC interacted with the PDZ2 βB-βC loop, though an interaction was not observed in their structure [26]. This prediction was based on isothermal titration calorimetry experiments in which comparative binding affinities between the APC peptide and Dlg1 PDZ1 and PDZ2 were measured. The APC-PDZ1 K_d_ was measured to be 18.2+/−7.6 µM, while the APC-PDZ2 K_d_ was measured to be 1.05+/−0.22 µM. Zhang et al. hypothesized that the different affinities between APC and the two PDZ domains could be due to sequence differences in the respective βB-βC loops (Figure 2A). A glutamine resides at position 340 in PDZ2, while the equivalent residue in PDZ1 is a proline. When Zhang et al. made the PDZ2 Q340P mutation, the affinity for the APC peptide decreased, with a K_d_ equal to 9.80+/−1.11 µM, implicating a role for the βB-βC loop in the PDZ2-APC interaction. In the structure reported here, Q340 stabilizes the βB-βC loop, using its side chain to form hydrogen bonds with the G344 backbone amide and the N346 side chain (Figure 4D). The order Q340 confers to the βB-βC loop orients the N339 side chain towards the APC peptide, promoting its hydrogen bonding to the APC V2840 backbone amide and carbonyl. Additionally, the Q340 and N339 backbone carbonyls are oriented to interact with the APC Y2838 backbone. A proline substitution at residue 340 would prohibit these backbone interactions and restrict the ability of N339 to engage the APC peptide, thereby resulting in decreased affinity for APC. Dlg1 PDZ1 has a lower affinity for APC than Dlg1 PDZ2, and differences in the βB-βC loop may underlie this differential. The conformation of the APC peptide bound to Dlg1 PDZ1, also reported by Zhang et al., is similar to the APC peptide conformation observed in the structure reported here, bound to Dlg1 PDZ2 (Figure 4E) [26]. Differences in the orientation of the L2839 side chain are evident, as is a shift in the ultimate residue, V2843, emphasized by a positional change in the carboxylate group. The APC carboxylate group is closer to the βA-βB loop GLGF binding motif in the PDZ2 structure reported here than in the Dlg1 PDZ1-APC structure. A residue that may underlie this structural difference is the lysine residue K324 in the PDZ2 βA-βB loop, which is an arginine, R229, in PDZ1. The arginine guanidinium group alters the peptide-binding pocket in the vicinity of the APC carboxylate group, effectively drawing the APC peptide away from the PDZ1 GLGF motif. These conserved differences between Dlg1 PDZ1 and PDZ2, likely explain the domains’ differential affinities for APC.

### Ligand-dependent PDZ Clustering

**Figure 5:**
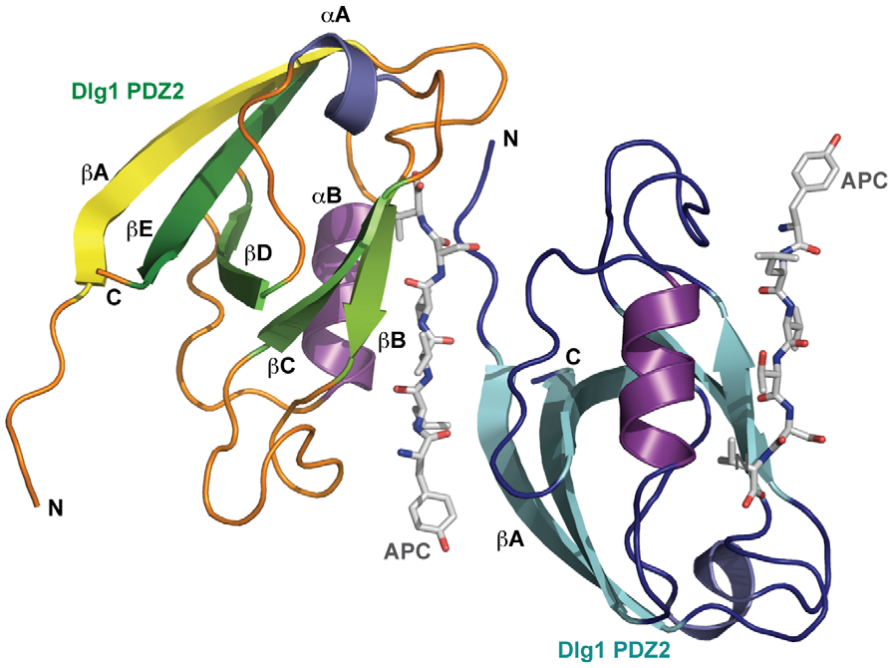
The Dlg1 PDZ2-APC complex mediates ligand-dependent interactions with a Dlg PDZ2 symmetry mate. Ribbon diagram of the Dlg1 PDZ2-APC complex with one complex colored as in Fig. 1C and a crystal symmetry mate colored with Dlg1 PDZ2 β-strands in cyan, helices αA and αB in teal and purple, and loops in dark blue. The Dlg1 PDZ2 symmetry mate uses its N-terminal loop and βA strand to interact with the APC peptide from the symmetry mate, potentially reflecting a ligand-dependent PDZ clustering mechanism.

PDZ domain-containing proteins, including the MAGUK family, often have PDZ domain arrays. In Dlg1, PDZ1 and PDZ2 are immediately adjoined in sequence, while PDZ3 is separated from PDZ2 by a 49 amino acid linker. Studies investigating the spatial arrangement of PDZ domains have observed distinct structural states between tandem PDZ domains, with changes inherent upon peptide binding [16], [17], [18], [19], [20], [21], [22]. To investigate potential higher order PDZ-PDZ interactions, I examined the symmetry mate interactions in the P2_1_2_1_2_1_ crystal lattice. Dlg1 PDZ2-APC forms an extensive interaction with a symmetry-related complex utilizing the APC peptide in one complex as a prime binding-determinant. The N-terminal PDZ2 βA strand from a symmetry mate binds along the length of the neighbouring APC peptide (Figure 5). This interaction between symmetry mates may mimic ligand-dependent PDZ clustering interactions in MAGUK proteins. Because PDZ1 and PDZ2 are immediately adjoined in sequence, the observed symmetry mate interaction would not be possible between PDZ1 and PDZ2 given the location of their respective C- and N-termini. However, the linker that spans PDZ2 and PDZ3 would enable these PDZ domains to potentially cluster via a ligand-dependent interaction as observed here.

## Discussion

Dlg1 PDZ2 conforms to a common G–H class PDZ domain (G335, H384) with class I peptide-binding activity [15]. The APC C-terminal sequence YLVTSV is a type I sequence (S/T-x-φ) that engages the βB - αB furrow, forming standard hydrogen bonds between the APC C-terminal carboxylate group and the backbone amides of the βA-βB loop while the ultimate APC side chain packs against hydrophobic residues in the PDZ furrow: L329, F331 and L391. Comparison of the Dlg1 PDZ2-APC complexes determined here and by Zhang et al. provide insight into binding plasticity and potential pathways involved in binding [26]. In the Zhang et al. structure, the APC peptide engages PDZ with its ultimate four residues while the preceding two residues are splayed away from the PDZ domain in a bent conformation (Figure 4C). When compared to the structure presented here which is more akin to a straight peptide conformation, only the ultimate APC valine residues align. All other residues are positioned in different rotamer conformations and the backbone of the two APC peptides are angled 45° relative to one another (Figure 4C). The structure of the APC peptide bound to PDZ2 reported here, conforms more to the peptide arrangement observed in the Dlg1 PDZ1-APC structure determined by Zhang et al. (Figure 4E) [26]. The difference between the two Dlg1 PDZ2-APC structures may reflect sequential binding steps. The Zhang et al. structure may represent an initial binding mode delineated by an interaction between the ultimate APC valine residue and the PDZ domain. This initial interaction may then transit to a higher-order engagement as additional conserved APC residues bind the PDZ2 βB-αB furrow and the βB-βC loop. These dual structural states support the previous observation of a sequential binding mechanism for the Dlg PDZ2 domain [51]. The straight APC conformation reported here and the additional PDZ2 contacts observed in that conformation may reflect a higher-affinity bound state. In support, the conformation affirms a prediction posed by Zhang et al. that the APC peptide would make additional contacts with the βB-βC loop based on the reduced affinity a PDZ2 βB-βC loop mutant had for APC peptide binding [26]. In addition, the straight APC peptide conformation observed in our structure is highly similar to the straight conformation observed in the Dlg1 PDZ1-APC structure, also determined by Zhang et al. [26]. Finally, the conformation of the PDZ-binding furrow in the Zhang et al. Dlg1-PDZ2-APC structure is more homologous to the closed state observed in the peptide-free Dlg1 PDZ2 apo structure, while the PDZ2 peptide furrow in the complex reported here is opened and engages additional determinants on the APC peptide.

How PDZ arrays regulate protein-protein interactions by sensing and clustering the proteins they interact with has remained an outstanding question. To investigate how PDZ domains might promote interactions between bound targets, I investigated whether residues that line the PDZ binding furrow are conserved with the hypothesis that a peptide-bound PDZ domain could form a composite binding site for protein-protein interactions. Across Dlg PDZ domains, core residues that line the peptide-binding furrow, involved in peptide binding, are conserved. Surrounding this region is a rim of conserved residues specific to Dlg1-PDZ2, flanked by conserved regions specific to species whose APC protein conforms to the invariant ultimate sequence GSYφVTSV. These concentric, conserved sub-regions are unique to the peptide binding face of the PDZ domain and are not maintained on the non-peptide binding face. These residues may support the architecture of the binding furrow, and/or they may act with the bound peptide to form a composite binding site. PDZ intra-molecular interactions have been observed to change, dependent on the presence or absence of bound peptide. Whether APC-bound PDZ2 forms a composite binding site for other PDZ domains and/or their binding partners warrants further investigation. Potential insight into peptide-dependent PDZ clustering comes from observed crystal packing interactions between symmetry mates in which the βA strand in a neighboring PDZ2 domain interacts with the APC peptide in an anti-parallel orientation.

The interaction observed between Dlg1 PDZ2 and APC facilitates a link between a cortical polarity determinant and the APC signaling scaffold. APC can interact directly with the microtubule cytoskeleton through a C-terminal basic region, or indirectly through its C-terminal EB1 SxIP binding motif that confers EB1-dependent APC microtubule plus end tracking activity [40], [41], [42]. It is of note that the EB1 binding site and the PDZ binding site are proximal to one another, separated by 30 amino acids. Whether there is structural communication between these two sites remains to be determined. Collectively, the structure presented here highlights the molecular determinants that mediate the Dlg1 PDZ2-APC interaction and facilitate stable, polarized microtubule attachment to a cortical polarity determinant and contribute to the molecular bases that underlie directed cell migration [6], [52].
